# Complications and Recurrence After Pelvic Exenteration for Gynecologic Malignancies: Survival Analysis From the COREPEX Study

**DOI:** 10.1097/AOG.0000000000006051

**Published:** 2025-09-11

**Authors:** Nicolò Bizzarri, Denis Querleu, Giulio Ricotta, Diana Giannarelli, Mihai Emil Cãpîlna, Santiago Domingo, Vito Chiantera, Hüseyin Akıllı, David Cibula, Zoltan Novák, Diana Zach, Andrea Miranda, Porfyrios Korompelis, Enrique Chacon, Ignacio Zapardiel, Björn Lampe, Valentyn Svintsitskyi, Olga Matylevich, Gabrielle H. van Ramshorst, Cagatay Taskiran, Fuat Demirkıran, Tibor Lengyel, Giuseppe Vizzielli, Matteo Loverro, Gwenael Ferron, Alejandra Martinez, Elodie Gauroy, Emmanuel Ladanyi, Szilard Leo Kiss, Victor Lago, Manel Montesinos-Albert, Mariano Catello Di Donna, Giuseppe Cucinella, Ali Ayhan, Jiri Slama, Viktória Rosta, Sahar Salehi, Mustafa Zelal Muallem, Ali Kucukmetin, Giovanni Scambia

**Affiliations:** UOC Ginecologia Oncologica, Dipartimento di Scienze Della Salute Della Donna, Del Bambino e di Sanità Pubblica, and the Biostatistics Unit, Scientific Directorate, Fondazione Policlinico Universitario A. Gemelli, IRCCS, Rome, the Unit of Gynecologic Oncology, Istituto Nazionale Tumori–IRCCS Fondazione G. Pascale, Naples, and the Department of Medicine, University of Udine, and the Clinic of Obstetrics and Gynecology, “Santa Maria della Misericordia” University Hospital, Azienda Sanitaria Universitaria Friuli Centrale, Udine, Italy; the Department of Surgical Oncology, Department of Surgical Oncology and INSERM CRCT Team 19, Oncogenesis of Sarcomas, and the Department of Surgical Oncology and INSERM CRCT Team 1, Institut Universitaire du Cancer Toulouse Oncopole, Toulouse, France; the First Obstetrics and Gynecology Clinic, “G. E. Palade” University of Medicine, Pharmacy, Science and Technology, Targu Mures, Romania; the Department of Gynecologic Oncology, La Fe University and Polytechnic Hospital, the POG Department University of Valencia, and the CEU Cardenal Herrera University, Valencia, Gynecologic Oncology, Universidad de Navarra, Pamplona, and the Gynecologic Oncology Unit, La Paz University Hospital, Madrid, Spain; the Division of Gynecologic Oncology, Department of Obstetrics and Gynecology, Başkent University Hospital, Ankara, and the Department of Obstetrics and Gynecology, Koc University School of Medicine and VKV American Hospital, and the Division of Gynecologic Oncology, Department of Obstetrics and Gynecology, Cerrahpasa Faculty of Medicine, Istanbul University, Istanbul, Turkey; the Department of Gynaecology, Obstetrics and Neonatology, First Faculty of Medicine, Charles University and General University Hospital in Prague, Prague, Czech Republic; the Department of Gynaecology, National Institute of Oncology, Budapest, Hungary; the Department of Pelvic Cancer and Women's and Children's Health, Karolinska University Hospital and Karolinska Institutet, Stockholm, Sweden; the Department of Gynecology with Center for Oncological Surgery, Charité Universitätsmedizin Berlin, corporate member of Freie Universität Berlin, Humboldt-Universität zu Berlin, and Berlin Institute of Health, Virchow Campus Clinic, Charité Medical University, Berlin, and the Department of Gynecology and Obstetrics, Florence-Nightingale-Hospital, Düsseldorf, Germany; the Northern Gynaecological Oncology Centre (NGOC), Queen Elizabeth Hospital, Gateshead, United Kingdom; the National Cancer Institute, Kiev, Ukraine; the NN Alexandrov National Cancer Centre of Belarus, Minsk, Belarus; the Department of Gastrointestinal Surgery, University Hospital Ghent, and Human Structure and Repair, Ghent University, Ghent, Belgium; and the National Cancer Institute, Bratislava, Slovakia.

## Abstract

**OBJECTIVE:**

To collect data from patients undergoing pelvic exenteration in recent clinical practice. The primary aim was 5-year disease-free survival. Secondary aims were 5-year overall survival, patterns of recurrence, identification of subgroups at higher risk of recurrence and death, survival associated with lymph node metastasis, and development of a prognostic score.

**METHODS:**

This was a retrospective, multicenter, international study conducted in tertiary national gynecologic oncology referral centers. Inclusion criteria included cervical, vaginal, vulvar, or endometrial cancer; anterior or total pelvic exenteration performed between January 2005 and March 2023; curative or palliative intent; and with or without laterally extended endopelvic or pelvic resection. Patients were excluded if they underwent posterior pelvic exenteration only or if preoperative computed tomography (CT), positron emission tomography (PET)–CT, or PET was not performed. A prognostic score was developed that was based on multivariable analysis.

**RESULTS:**

Eight hundred sixty-two patients were included. Surgical margins were tumor free in 676 (78.4%). In patients treated with curative intent, total pelvic exenteration, positive surgical margins, and presence of lymphovascular space invasion were independently associated with worse disease-free survival. Performance of lymphadenectomy was associated with better disease-free survival. Total pelvic exenteration, positive surgical margins, and presence of lymphovascular space invasion were factors independently associated with decreased overall survival. Performing pelvic exenteration at time of persistent (instead of recurrent) disease negatively affected overall survival. Prognostic score identified four risk groups with a 5-year disease-free survival of 43.7%, 24.9%, 22.2%, and 8.0% (*P*<.001). The 5-year overall survival in the four risk groups was 54.3%, 40.4%, 24.0%, and 4.3% (*P*<.001). The most frequent sites of recurrence were distant in 166 patients (32.1%). The 5-year disease-free survival and cancer-specific survival in patients with para-aortic lymph node metastasis were significantly worse compared with those in patients with pelvic-only metastatic nodes or with negative nodes (*P*=.002 and *P*<.001, respectively).

**CONCLUSION:**

Independent factors associated with worse disease-free survival and overall survival and subgroups of patients at higher risk of recurrence and death were identified. A multivariable prognostic score was developed that can be used for patient counseling and surveillance strategies and for future prospective studies.

## Abstract 2

Subgroups of patients at higher risk of recurrence and death were identified and a prognostic score was developed in a large cohort undergoing pelvic exenteration for gynecologic cancers.

Pelvic exenteration is a radical operation that involves the en bloc resection of the pelvic organs, including the genital, urinary tract, and bowel. It is accompanied by significant perioperative morbidity, important psychologic and quality-of-life effects, and high costs of hospitalization.^1–4^ Pelvic exenteration is used as salvage option to treat patients with pelvic recurrence or persistence of gynecologic cancers after radiotherapy or as primary treatment for selected cases.^1,5,6^ In patients in whom this surgical procedure is performed with curative intent, overall survival can reach 60% at 5 years.^7–9^ Nevertheless, a proportion of women still experience recurrence shortly after the pelvic exenteration.^6–10^ In palliative settings, pelvic exenteration has a role in improving quality of life, with 5-year survival reported to be 10–27%.^10,11^ Patient selection is thus imperative. However, anterior–total pelvic exenteration is a rare procedure, and most of the data reported to date are from single centers with relatively small numbers of patients or heterogeneous groups of patients; therefore, the ability to draw clinically relevant conclusions on oncologic outcomes is reduced.^1,6–9^ Moreover, the majority of reports were published before the introduction of high-performance preoperative imaging techniques, such as pelvic magnetic resonance imaging (MRI), positron emission tomography (PET)–computed tomography (CT), and modern radiotherapy.^1,6–9^

With the COREPEX study (COmplications and REcurrence after PElvic eXenteration for gynecologic malignancies), we established a database reflecting contemporary clinical practice of pelvic exenteration in gynecologic malignancies. Our objectives were to investigate the effect of pelvic exenteration in gynecologic malignancies on oncologic outcomes, to identify prognostic factors, to analyze differences in recurrence patterns, and to develop a prognostic score.

## METHODS

This was an international, multicenter, retrospective study. The COREPEX study consortium consisted of 20 tertiary cancer centers from Europe. Imaging modalities used for clinical staging included MRI or expert ultrasonography (ultrasound scan performed by a physician with expertise in gynecologic oncology) and CT or PET–CT scan.

Requirements for a center to join the study were as follows: availability of imaging modalities for preoperative workup and follow-up (MRI or expert ultrasonography, CT, PET–CT, PET scan); national referral centers for gynecologic oncology (tertiary referral unit with specialist expertise in gynecologic oncology surgery); all pelvic exenteration cases discussed by multidisciplinary teams in both the preoperative and postoperative periods; availability of a prospectively collected database of cases; surgery performed by experienced gynecologic oncologists; pathology performed by a dedicated gynecologic oncology pathologist; and institutional follow-up performed by physicians.

Patients were included if they met the following inclusion criteria: histologically confirmed cervical, vaginal, vulvar, or endometrial cancer; anterior or total pelvic exenteration performed between January 2005 and March 2023; curative or palliative intent; and with or without laterally extended endopelvic resection^12^ and laterally extended pelvic resection.^13^ Patients were excluded if they underwent posterior pelvic exenteration only or if a preoperative CT, PET–CT, or PET was not performed. *Total pelvic exenteration* was defined as the removal of the uterus (when present), vagina, urethra, bladder, and rectum; *anterior pelvic exenteration* involved the same procedures without rectal resection.^1^

The primary aim of this study was 5-year disease-free survival. Secondary outcomes were 5-year overall survival, pattern of recurrence, definition of subgroups at higher risk of recurrence and death, survival associated with lymph node metastasis, and development of a prognostic score.

The protocol was approved by the IRB of the lead institution (Fondazione Policlinico Agostino Gemelli IRCCS, Rome, Italy) on March 25, 2021 (protocol 3879, No. 0011322/21). The investigators obtained informed consent from each participant or each participant's guardian when required. The study was performed in accordance with the Declaration of Helsinki and followed the STROBE (Strengthening the Reporting of Observational Studies in Epidemiology) reporting guidelines.

Data were collected from the principal investigator at each institution and entered in the COREPEX study electronic database using RedCap software. The database is held by Policlinico Agostino Gemelli IRCCS, Rome, Italy. The included patients' data were pseudonymized in the COREPEX database, and the code key was kept at each institution according to good clinical practice and local regulations.

Clinicopathologic variables included age at pelvic exenteration, body mass index (BMI, calculated as weight in kilograms divided by height in meters squared), Eastern Cooperative Oncology Group performance status, primary site of disease, histology, PET or PET–CT, previous radiotherapy, time from diagnosis to pelvic exenteration, pelvic exenteration intent (curative vs palliative), timing of pelvic exenteration (naive, *persistence* [defined as lack of complete remission of the tumor in its primary site], *recurrence* [defined as cancer that has come back after a period of time during which the cancer could not be detected]), type of pelvic exenteration (anterior only or total), pelvic exenteration approach (laparotomy, laparoscopic, robot, or conversion), laterally extended surgery (laterally extended endopelvic resection,^12^ laterally extended pelvic resection^13^), pelvic exenteration classification (supralevator, infralevator, infralevator with vulvectomy),^14^ lymphadenectomy (pelvic, para-aortic, inguinal), length of hospitalization, metastatic lymph node(s), surgical margins, pathologic tumor diameter, lymphovascular space invasion, perineural invasion, and adjuvant chemotherapy.

Demographics and clinical data were summarized as absolute counts and percentages for categorical variables and median values and interquartile range for continuous variables. *Disease-free survival* was defined as the time interval between the date of pelvic exenteration and the evidence of the first disease recurrence or death resulting from disease. *Overall survival* was defined as the time interval between the date of pelvic exenteration and date of death resulting from any cause. *Cancer-specific survival* was defined as the time interval between the date of pelvic exenteration and the date of death resulting from cancer. Intervals were censored at the date of last follow-up if no event was observed. We used the Kaplan–Meier method to estimate the distribution of time-to-event endpoints of disease-free survival and overall survival and assessed differences among curves with the log-rank-test.^15,16^ Univariable and multivariable analyses were performed with the Cox proportional hazard model, and hazard ratios (HRs) were reported with their 95% CIs.^17^ In univariable and multivariable Cox regression analyses for disease-free survival, both recurrence–progression and deaths were considered as events. To enter Cox regression analysis, continuous variables were categorized with cutoff values based on clinical relevance. Only variables that were significant (*P*<.05) in univariable analysis were selected for multivariable analysis. A stepwise selection based on likelihood ratio was used to select variables in the multivariable approach with values of *P*=.05 and *P*=.10 to enter and remove limits, respectively. With this approach, at each step, the most significant variable (the lowest *P* value) enters the model, and the significance level for all variables is recalculated; the entry level was *P*<.05, and, if, after other factors are added, one of the variables reaches *P*>.10, it is removed. The Harrell concordance statistics factor was used to assess the discrimination power of each model. A score was built reproportioning the regression coefficients to sum up to 100% using the β coefficients derived from each final model. In detail, the β coefficients from the multivariable model (statistical coefficients representing the influence of each parameter) were converted into risk points to create a scoring system. This scoring system is used to quantify the risk associated with each parameter for an individual patient; from these risk points, patients were divided into risk groups with significantly different prognoses for survival. Four groups were considered that were based on risk score (0–25%, 26–50%, 51–75%, 76–100%), and Kaplan–Meier curves for each group were calculated.

Subgroup survival analyses according to site of disease were conducted, and stratification was performed according to risk factors identified by literature references^1,6–10^ and results from multivariable analysis of the present study.

The pattern of recurrence was calculated in patients who experienced relapse (all patients with recurrence were included, as well as those operated with palliative intent). In the multivariable analysis stratified according to site of recurrence, only recurrences were considered as events. Survival analysis performed according to site of disease and risk factors for site of recurrence included the variables identified in literature references.^1,6–10^ IBM SPSS 27.0, R 4.1.2, and library “survival” and “survminer” were used.

## RESULTS

**Fig. 1.**
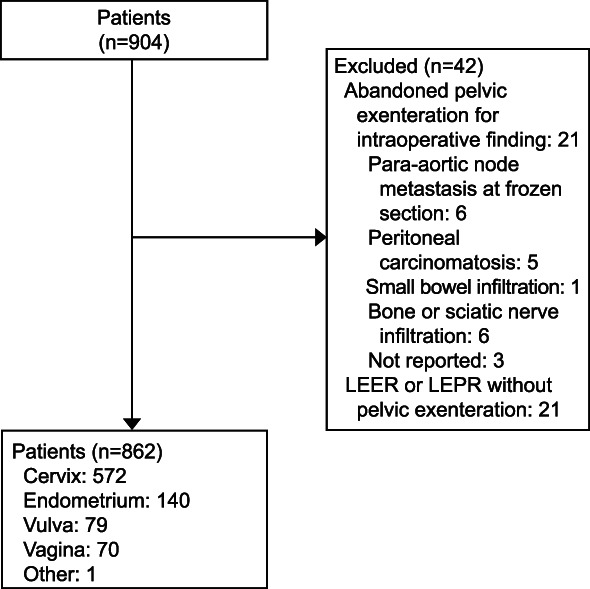
Inclusion and exclusion flowchart. LEER, laterally extended endopelvic resection; LEPR, laterally extended pelvic resection.

Data from 904 patients were retrieved. Of these, 21 (2.3%) were excluded because pelvic exenteration was abandoned as a result of intraoperative findings of distant metastasis and 21 (2.3%) were excluded because they underwent laterally extended endopelvic resection or laterally extended pelvic resection with no pelvic exenteration, leaving a total of 862 patients included in the analysis (Fig. 1). Table 1 shows the clinical and pathologic characteristics of the included patients. The majority of patients were diagnosed with cervical cancer (n=566, 65.7%) and squamous cell carcinoma (n=581, 67.4%). A PET or PET–CT scan was performed in 523 patients (60.7%), and the median time from diagnosis to pelvic exenteration was 17 months (interquartile range 8.5–40.1 months). Most of pelvic exenterations were performed with curative intent (n=759, 88.1%), at the time of recurrence (n=648, 75.2%), and by laparotomy (n=779, 90.4%). Total pelvic exenteration was performed in 510 cases (59.2%) and infra-levator pelvic exenteration in 343 cases (39.8%). Laterally extended endopelvic resection or laterally extended pelvic resection was associated with pelvic exenteration in 193 patients (22.4%). One hundred twenty-one patients (14.0%) and 53 patients (6.1%) had metastatic pelvic and para-aortic lymph nodes, respectively. Surgical margins were free from tumor in 676 patients (78.4%). A total of 198 patients (23.0%) received adjuvant chemotherapy.

*Bizzarri. Survival After Pelvic Exenteration for Gynecologic Malignancies. Obstet Gynecol 2025*.

**Table 1.**
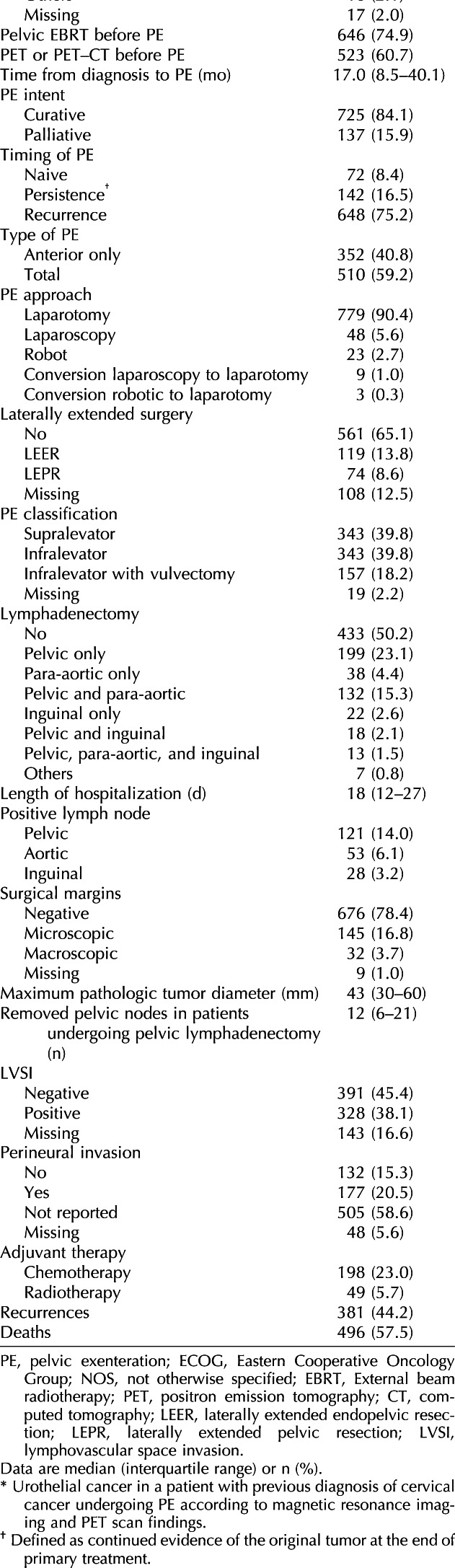
Clinical and Pathologic Characteristics of the Included Patients (N=862)

Median follow-up time was 47 months (interquartile range 20–87 months). Overall, 381 patients (44.2%) had a recurrence, 496 (57.5%) died of any cause, and 379 (44.0%) died of disease. No patient experienced intraoperative death, and 27 patients (3.1%) died of postoperative complications within 30 days (Appendix 1, available online at http://links.lww.com/AOG/E303). The 3- and 5-year disease-free survival rate in the entire population was 28.7% (95% CI, 25.2–32.2%) and 24.3% (95% CI, 20.8–27.8%), respectively. The 3- and 5-year overall survival was 37.3% (95% CI, 33.6–41.0%) and 30.3% (95% CI, 26.4–34.2%), respectively; the 3- and 5-year cancer-specific survival was 44.8% (95% CI, 40.7–48.9%) and 38.2% (95% CI, 33.9–42.5%), respectively (Appendix 2, available online at http://links.lww.com/AOG/E303).

Table 2 shows the univariable and multivariable analyses associated with reduced disease-free survival and overall survival in the group of patients treated with curative intent (intended as patients with no metastatic para-aortic or inguinal lymph nodes, no peritoneal carcinomatosis, n=725). In the adjusted analysis, total pelvic exenteration (HR 1.43, 95% CI, 1.14–1.80, *P*=.002), tumor-involved surgical margins (HR 1.82, 95% CI, 1.40–2.37, *P*<.001), and presence of lymphovascular space invasion (HR 1.58, 95% CI, 1.27–1.98, *P*<.001) were factors independently associated with worse disease-free survival. In addition, performing lymphadenectomy was an independent factor associated with improved disease-free survival (HR 0.80, 95% CI, 0.64–0.99, *P*=.049).

**Table 2.**
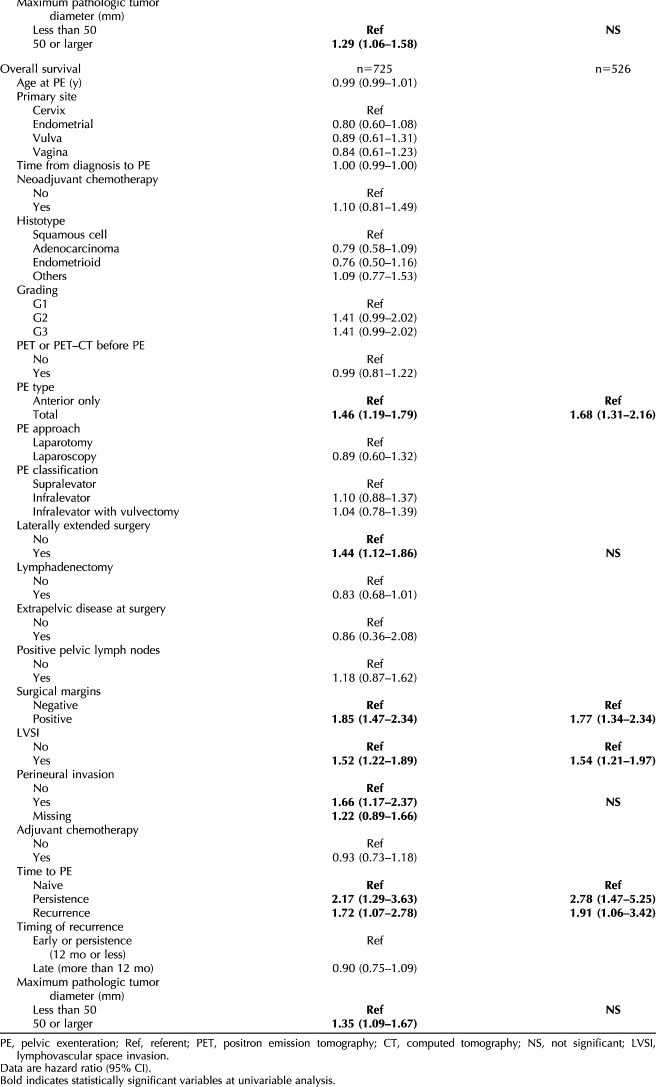
Univariable and Multivariable Analyses for Risk of Recurrence and Death in Patients Treated With Curative Intent

Total pelvic exenteration (HR 1.68, 95% CI, 1.31–2.16, *P*<.001), tumor-involved surgical margins (HR 1.77, 95% CI, 1.34–2.34, *P*<.001), and presence of lymphovascular space invasion (HR 1.54, 95% CI, 1.21–1.97, *P*=.001) were factors independently associated with worse overall survival. Moreover, performing pelvic exenteration at the time of persistence (instead of at recurrence or in the primary setting) represented an independent factor negatively affecting overall survival (HR 2.78, 95% CI, 1.47–5.25, *P*=.002).

Appendix 3 (available online at http://links.lww.com/AOG/E303) demonstrates the survival (disease-free, overall, and cancer-specific survival) associated with site of disease (cervical, endometrial, and vulvar or vaginal cancer) according to the presence of major risk factors.

**Fig. 2.**
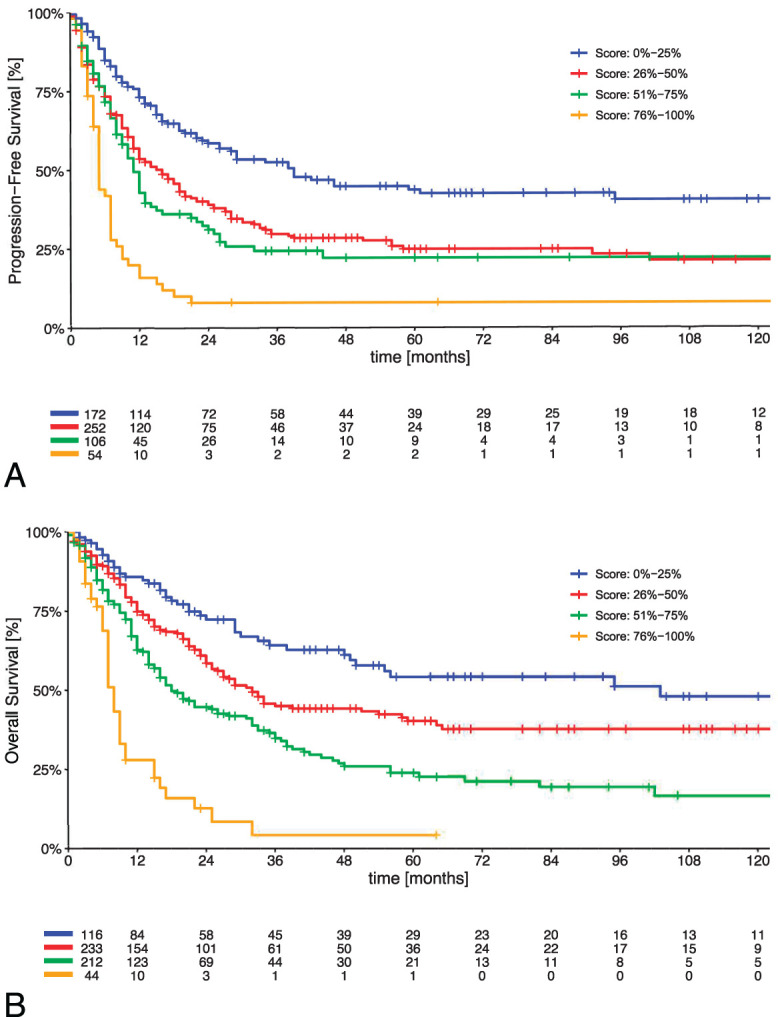
Multivariable prognostic score defining risk groups associated with different disease-free **(A)** and overall survival **(B)**. **A.** Overall log-rank test *P*<.001; 0–25% vs 26–50% *P*<.001; 26–50% vs 51–75% *P*=.28; 51–75% vs 76–100% *P*<.001. **B.** Overall log-rank test *P*<.001; 0–25% vs 26–50% *P*=.016; 26–50% vs 51–75% *P*<.001; 51–75% vs 76–100% *P*<.001. Prognostic score is calculated according to Table 3 (eg, a patient who underwent total pelvic exenteration with lymphadenectomy and had positive lymphovascular space invasion with negative surgical margins has a score of 22+26=48; the patient falls into the second risk group, with a 5-year disease-free survival of 24.9% [red group in Fig. 2A]).

The prognostic model for disease-free survival consisted of four parameters: 1) surgical margins, 2) lymphovascular space invasion, 3) type of pelvic exenteration, and 4) lymphadenectomy (Table 3 and Fig. 2A). The β coefficients of the multivariable model were consequently converted into risk points (Table 3 and Fig. 2A). The Harrell concordance statistic factor (C statistics) of the resulting model for disease-free survival was 0.63 (95% CI, 0.60–0.66). The Kaplan–Meier disease-free survival curve for the risk-score groups is shown in Figure 2A. Four risk groups significantly differing in prognosis were identified with a 5-year disease-free survival of 43.7% (95% CI, 35.1–52.3%) for scores of 0–25%, 24.9% (95% CI, 18.4–31.4%) for scores of 26–50%, 22.2% (95% CI, 13.0–31.4%) for scores of 51–75% and 8.0% (95% CI, 0–15.4%) for scores of 76–100% (*P*<.001).

**Table 3.**
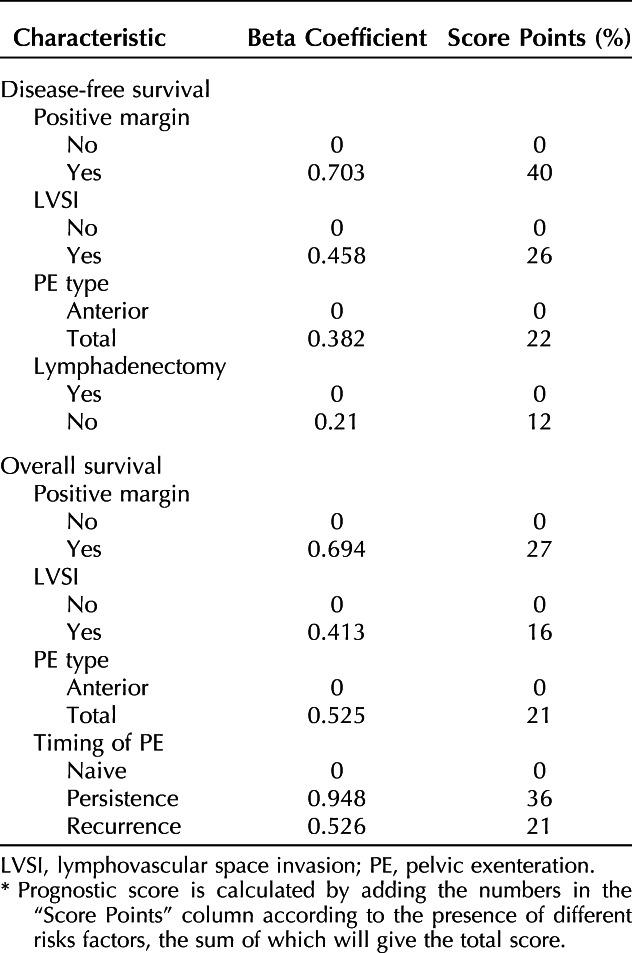
Prognostic Score Model for Disease-Free Survival and Overall Survival*

*Bizzarri. Survival After Pelvic Exenteration for Gynecologic Malignancies. Obstet Gynecol 2025*.

The prognostic model for overall survival consisted of four parameters: 1) surgical margins, 2) lymphovascular space invasion, 3) type of pelvic exenteration, and 4) timing of pelvic exenteration (Table 3 and Fig. 2B). The β coefficients of the multivariable model were consequently converted into risk points (Table 3 and Fig. 2B). The Harrell concordance statistic factor (C statistics) of the resulting model for disease-free survival was 0.66 (95% CI, 0.63–0.68). The Kaplan–Meier overall survival curve for the risk-score groups is shown in Figure 2B. The 5-year overall survival in the four risk groups was 54.3% (95% CI, 43.1–65.5%) for scores of 0–25%, 40.4% (95% CI, 32.6–48.2%) for scores of 26–50%, 24.0% (95% CI, 16.7–31.2%) for scores of 51–75%, and 4.3% (95% CI, 0–12.1%) for scores of 76–100% (*P*<.001).

**Fig. 3.**
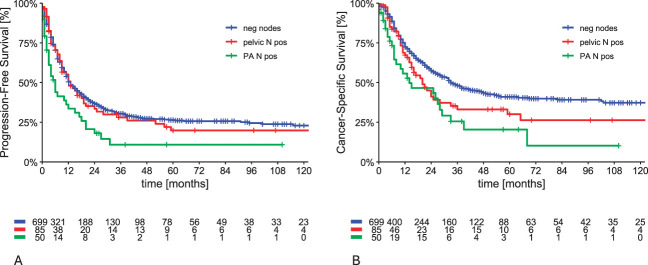
Disease-free survival **(A)** and cancer-specific survival **(B)** associated with lymph node (N) metastasis. PA, para-aortic.

In the entire cohort of patients, those with any metastatic lymph node (pelvic, para-aortic, inguinal, n=163) had significantly worse 5-year disease-free survival (26.6% [95% CI, 22.7–30.5%] vs 15.1% [95% CI, 7.5–22.7%], *P*=.020) and cancer-specific survival (41.2% [95% CI, 36.3–46.1%] vs 27.3% [95% CI, 17.5–37.1%], *P*<.001) compared with those with negative lymph nodes (n=699). When patients with metastatic inguinal nodes were excluded (leaving n=834 patients), the 5-year disease-free survival in patients with para-aortic lymph node metastasis, 10.8% (95% CI, 0.2–21.4%), was significantly worse compared with patients with pelvic-only metastatic nodes, 18.2% (95% CI, 8.0–28.4%), or with negative nodes, 26.6% (95% CI, 22.7–30.5%) (overall *P*=.002, para-aortic vs others *P*<.001) (Fig. 3A). Similarly, the 5-year cancer-specific survival in patients with para-aortic lymph nodes metastasis, 20.8% (95% CI, 5.3–36.3%), was significantly worse compared with patients with pelvic-only metastatic nodes, 30.0% (95% CI, 17.5–42.5%) or with negative nodes, 41.2% (95% CI, 36.3–46.1%) (overall *P*<.001, para-aortic vs others *P*=.006) (Fig. 3B). Patients with metastatic inguinal lymph nodes (n=28) (with or without other lymph node involvement) had significantly worse 5-year disease-free survival and cancer-specific survival (16.1% [95% CI, 1.6–30.6%] and 11.5%, [95% CI, 0–31.1%]) than patients with pelvic-only metastatic nodes or with negative nodes (*P*=.007 and *P*=.013, respectively).

*Bizzarri. Survival After Pelvic Exenteration for Gynecologic Malignancies. Obstet Gynecol 2025*.

**Fig. 4.**
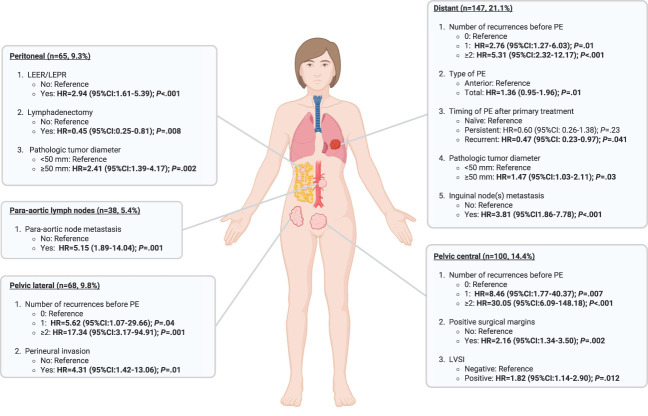
Multivariable model showing risk factors specifically associated with different sites of recurrence. Total number of patients 696 (patients without missing data on variables listed in multivariable analysis). Image created with BioRender. LEER, laterally extended endopelvic resection; LEPR, laterally extended pelvic resection; HR, hazard ratio; PE, pelvic exenteration; LVSI, lymph vascular space involvement.

A total of 381 patients (44.2%) experienced relapse. Sites of recurrence (total sites of recurrences 517; one patient could have had more than one site of recurrence at the same time) were distant in 166 (32.1%), pelvic central in 119 (23.0%), peritoneal in 87 (16.8%), pelvic lateral in 83 (16.1%), para-aortic in 43 (8.3%), and unknown in 19 (3.7%). Risk factors specifically associated with different site of recurrence are reported in a multivariable model shown in Figure 4 and Appendix 4 (available online at http://links.lww.com/AOG/E303). Recurrences were treated with chemotherapy in 166 (43.6%), surgery in 34 (8.9%), radiotherapy in 28 (7.3%), no treatment in 110 (28.9%), and other treatments in 43 (11.3%).

*Bizzarri. Survival After Pelvic Exenteration for Gynecologic Malignancies. Obstet Gynecol 2025*.

## DISCUSSION

With this study, we showed that 5-year disease-free survival, overall survival, and cancer-specific survival in patients undergoing total or anterior pelvic exenteration for gynecologic cancers were 24.3%, 30.3%, and 38.2%, respectively. Relapse occurred in 44.2% of patients, the majority of whom had distant recurrence (32.1%) followed by central pelvic recurrence (23.0%). Risk factors independently associated with worse oncologic outcome were performance of total pelvic exenteration, positive surgical margins, lymphovascular space invasion, not performing lymphadenectomy, and performing pelvic exenteration in patients with persistent disease. Moreover, patients with para-aortic lymph node metastasis had worse survival outcomes compared with those with no or pelvic-only lymph node metastasis. Risk factors for specific site of recurrence were identified. Finally, we were able to develop a prognostic score model to predict the risk of recurrence and death.

In our study, we present survival outcomes comparable with outcomes from other series,^18–20^ and we show that, in patients treated with curative intent, surgical margin status, type of pelvic exenteration, and lymphovascular space invasion were the only factors independently affecting both disease-free survival and overall survival; pelvic lymph node metastasis did not significantly affect survival. Moreover, performing lymphadenectomy reduced the risk of recurrence. This finding could be explained by the therapeutic effect of the removal of macroscopic and occult nodal disease. In addition, total pelvic exenteration (vs anterior) was associated with better survival. We might postulate that leaving the posterior compartment during anterior pelvic exenteration could be associated with potential local residual or skip microscopic disease (despite negative margins), leading to recurrence. Furthermore, we assessed the prognostic value of para-aortic node metastases in a separate analysis because patients with para-aortic metastases were not included in the univariable and multivariable analyses (only patients treated with curative intent were selected for these models). Our results confirm that para-aortic node metastasis represents a negative prognostic factor, validating that pelvic exenteration should not be considered a curative approach in these patients, corroborating previous reports.^7^ In contrast to other studies, our findings showed that histology type and site of disease did not influence oncologic outcomes.^21,22^

With the present study, we developed a prognostic score (COREPEX score) to predict the risk of recurrence and death based on the identified risk factors independently associated with worse disease-free survival and overall survival. The score can be used in clinical practice to discuss with patients the risk of adverse oncologic outcomes and possibly to tailor adjuvant or maintenance therapy and surveillance strategy. The identification of risk factors specific for each site of recurrence can also be helpful for selecting patients for chemotherapy after pelvic exenteration and to adapt the surveillance with targeted imaging. Patients carrying independent risk factors for distant, peritoneal, or para-aortic node recurrence might be appropriate candidates for adjuvant therapy. To the best of our knowledge, this is the first study to report factors independently associated with different patterns of recurrence after pelvic exenteration.^19,20^ We reported 8.4% of patients undergoing pelvic exenteration as primary surgery. This is reported in selected cases, particularly for symptom control (heavy bleeding, fistula, bowel obstruction) or for tumors that cannot be irradiated with radical intent (large volume).

This study has several strengths. The first is that this is the largest international collaboration involving tertiary referral units with expertise in gynecologic oncology surgery, and the inclusion time frame was recent. A similar collaboration was developed earlier by the PelvEx collaborative group, which published different international studies on the outcomes of pelvic exenteration performed for all pelvic cancers.^23,24^ The PelvEx collaboration published a study on factors associated with outcomes after pelvic exenteration for advanced nonrectal pelvic malignancy (n=523 had gynecologic malignancies) and showed that having negative surgical margins was the main factor associated with survival.^24^ However, of gynecologic patients included in that study, 42.8% had ovarian cancer; this was an exclusion criterion for our study because most patients with ovarian cancer undergo posterior pelvic exenteration (with different morbidity profile) and present with disseminated peritoneal carcinomatosis. Another strength is that we reported detailed information about clinicopathologic characteristics, treatment, and sites of recurrence (with consequent development of risk models for specific site of disease). Last, this study represents the first study developing a prognostic score, which can be used for discussion with patients. This study has a number of limitations such as the retrospective design with potential selection bias and time–trend bias and the heterogeneity of included patients, albeit only including gynecologic malignancies. The exclusion of posterior pelvic exenteration for nonovarian cancers can be considered a limitation of the study, with consequent lack of survival analysis between anterior–total and posterior pelvic exenteration; however, this allows emphasis on a large number of surgeries involving bladder removal, which has the largest effect on quality of life.^25^ The fact that different disease sites may show different results or prognostic factors (limiting the applicability in the general population with gynecologic cancers), the lack of information on perioperative morbidity (reported in a separate study by the same group), and the lack of external validation of the prognostic score model (which could have added value to the score) might be also considered limitations.

Survival after pelvic exenteration for gynecologic malignancies remains poor, and the risk of recurrence is high. Nevertheless, risk factors associated with oncologic outcomes were clearly identified. We have presented patterns of recurrence and risk factors for site of recurrence and suggested the COREPEX prognostic score, a multivariable model developed to predict the risk of recurrence and death. This can be used to improve patient selection, for patients counseling, for surveillance strategies, and for future prospective studies.
